# Diabetes May Modulate the Association Between Age and Optical Coherence Tomography Angiography Parameters: A Serial, Cross-Sectional Study

**DOI:** 10.3390/jpm16060286

**Published:** 2026-05-26

**Authors:** Abu Taha, Yi Stephanie Zhang, Chu Jian Ma, Jay M. Stewart

**Affiliations:** 1 Department of Ophthalmology, University of California, 490 Illinois Street, Floor 5, San Francisco, CA 94143, USA; 2 Department of Ophthalmology & Visual Sciences, University of California, Davis, Sacramento, CA 95817, USA; 3 Department of Ophthalmology, Zuckerberg San Francisco General Hospital and Trauma Center, San Francisco, CA 94158, USA; 4 Department of Ophthalmology, Massachusetts Eye and Ear Infirmary, Harvard Medical School, Boston, MA 02114, USA; 5 Department of Ophthalmology and Visual Sciences, University of Illinois-Chicago, Chicago, IL 60607, USA

**Keywords:** OCTA, age, diabetic retinopathy, interaction, personalized screening

## Abstract

**Purpose:** We investigated the association between age and retinal microvasculature parameters as measured by optical coherence tomography angiography (OCTA) and the modifying effect of diabetes status on this association. **Methods:** In this serial, cross-sectional study, 3 × 3 mm^2^ macular OCTA images were obtained from healthy adults and adults with diabetes mellitus (DM) with no diabetic retinopathy (DR) or with mild non-proliferative DR (NPDR). The parameters analyzed included foveal avascular zone (FAZ) area and perimeter, vessel density (VD), vessel length density (VLD), and flow index (FI) of the superficial capillary plexus (SCP) and deep capillary plexus (DCP). The associations between OCTA parameters and age were explored using multivariable linear regression models. **Results:** For the included 1855 patients (1855 eyes) (49% male; mean age: 55 years), the results were as follows: no diabetes (N = 217), DM no DR (N = 1352), and mild NPDR (N = 286). Increasing age was significantly associated with decreased SCP and DCP VD and VLD in the diabetic and non-diabetic groups. The slope of association between SCP and DCP FI and age in the diabetic patients was significantly different than that in the control patients. **Conclusions:** The strength of the association between aging and OCTA parameters differed significantly between the controls and those with early retinopathy, pointing to a potentially altered retinal vascular homeostasis secondary to diabetic pathophysiology. This finding offers insight into the early pathological biomarkers of DR and may guide early DR management for patients based on personalized risk scores.

## 1. Introduction

The retina is an extension of the interconnected neuronal networks of the central nervous system, supported by the choroidal and retinal vascular plexuses [1]. Neuronal and microvascular degradation with aging has been shown both in the brain and the retina through histopathological studies demonstrating reduced vascular density [2,3,4] and atrophy on non-invasive imaging such as magnetic resonance imaging [5]. In the retina, optical coherence tomography structure and angiographic (OCTA) imaging have shown that with aging, vessel density (VD) and perfusion density (PD) decrease in the superficial capillary plexus (SCP) and deep capillary plexus (DCP) in healthy adults [3,6,7,8]. An improvement in speed and image quality has increased OCTA‘s prevalence in recent years, making it a valuable biomarker for microvascular changes in the body due to aging and systemic diseases [9].

Emerging research is uncovering new associations between OCTA biomarkers and systemic diseases [9,10]. Several ongoing clinical trials investigating diabetic eye disease have incorporated various OCTA-derived metrics to evaluate diabetic retinopathy severity [11]. Diabetic retinopathy (DR) is a complication of diabetes, characterized by microangiopathy in the retina [11] with features of neurodegeneration [12]. Studies have used OCTA to characterize hallmark DR vascular alterations such as microaneurysms, neovascularization, and retinal non-perfusion areas [9,13]. The utility of OCTA parameters such as VD, VLD, and foveal avascular zone (FAZ) area in distinguishing the presence and severity of DR has been studied extensively [14,15,16,17,18,19,20,21,22,23]. In addition, the impact of sociodemographic factors such as sex, race, ethnicity, and socioeconomic status on OCTA metrics has also been investigated [24,25,26]. However, the association between aging and retinal microvascular alterations on OCTA in patients with DR remains under-researched [3,6,27,28,29,30]. As we consider building normative values for various OCTA parameters and conduct diabetic vasculopathy studies [31], it is crucial to understand how physiological processes such as aging may interact with the pathophysiology of DR, thus impacting the clinical interpretation of these parameters.

In this study, we explored the association between age and several OCTA parameters in non-diabetic and diabetic patients to examine how the presence of diabetes may alter the existing relationship between age and OCTA metrics. Our study aims to fill a research gap that explores how diabetic retinopathy, a common disease entity, interacts with demographic factors like aging, while controlling for several confounders. We utilize various OCTA parameters as proxies of retinal microvasculature health. Since OCTA continues to gain popularity as a clinical tool assessing the extent of microvasculature loss in retina, such knowledge may aid future researchers about important modifiers that may exacerbate features of diabetic retinopathy. Furthermore, as precision and personalized medicine gains momentum, the interaction between aging and DR may translate clinically into individualized clinical management plans based on patient composite risk scores.

## 2. Methods

This research was conducted in accordance with the tenets of the Declaration of Helsinki. The study was approved by the Human Research Protection Program (HRPP) at the University of California, San Francisco (UCSF), and the San Francisco Department of Public Health (approval number: #17-21703; approval date: 29 August 2024), granting a waiver of consent affirming that patient welfare would not be adversely affected by waiving informed consent given no subject contact.

### 2.1. Study Sample

For this retrospective, serial, cross-sectional, observational study, OCTA scans and medical records were acquired from Zuckerberg San Francisco General Hospital and Trauma Center (ZSFG). The purpose of a repeated cross-sectional design was to observe trends in retinal microvasculature at various time points from different subjects sampled from the same population [32]. Diabetic patients were recruited from the diabetic telemedicine screening program between 2018 and 2022. The authors of this study began collecting study participants’ medical records starting in March 2021 up until March 2022, including identifying information. Color fundus photography, visual acuity (VA) testing, and OCTA imaging were performed during the visits. Non-diabetic patients were recruited from the general optometry clinic and had were referred for various ophthalmological concerns such as dry eye and presbyopia, and they underwent the same imaging protocols as the diabetic patients. Patients were asked if they wanted to take additional images as part of an umbrella study. No other indications were present for the patients to obtain OCTA imaging. Patients with ocular co-morbidities affecting the retina, such as vascular occlusion, glaucoma, and vitreomacular disease, were excluded. Diabetic patients who had a prior history of ocular procedures such as intravitreal injections or laser were also excluded. Age, sex, duration of diabetes, hypertension status, and most recent HbA1c level were obtained by patient report and from a review of medical records. Patients with missing demographic or clinical information were excluded from the analysis. Patients were grouped into non-diabetic controls, DM with no DR, and mild non-proliferative diabetic retinopathy (NPDR) groups. The hospital’s reading center graded DR severity using the ETDRS grading protocol [33].

### 2.2. Image Acquisition

All participants underwent ultra-widefield fundus photography (Optos Daytona, Optos PLC, Dunfermline, UK) and OCTA scanning with a Cirrus^TM^ HD-OCT 5000 with AngioPlex OCT Angiography (Carl Zeiss Meditec, Dublin, CA, USA), with 3 × 3 mm^2^ macular scans captured from both eyes of the patients. The 3 × 3 mm^2^ scans were utilized because of their superior resolution of the perifoveal vasculature, thereby reducing the challenge of manually delineating the foveal avascular zone [34]. Each scan was comprised of 245 clustered B-scans, repeated 4 times. Only images with a signal strength index (SSI) of 8 or above, without motion artifacts or media opacities, and decentration of <20 microns from the foveal center were included in the analysis. Images were obtained for both eyes, but only one eye, the right eye by default, was included in the study. In addition to the SSI provided by the machine, all images were manually reviewed for quality and potential confounding ocular diseases before inclusion.

### 2.3. Image Analysis

The OCTA parameters were divided into three categories: foveal avascular zone-related metrics, consisting of foveal avascular zone (FAZ) area and perimeter; vessel density (VD) metrics, including VD and vessel length density (VLD); and perfusion-related metrics, namely, flow index (FI).

FAZ area and perimeter were calculated with ImageJ (1.53J) (U.S. National Institutes of Health, Bethesda, MD, USA), using the tracing tool to delineate the FAZ on the whole retina angiograms that included both the SCP and DCP [35]. Manual grading of the FAZ parameters in ImageJ was completed by two separate graders (A.T.T. and Y.S.Z.) for 10% of the images to confirm intergrader reliability before grading the rest of the sample set.

The SCP and DCP were derived from the inner retina angiograms, with SCP making up the inner 70% and DCP comprising the outer 30% of the inner retinal thickness. The inner retina was defined as an offset distance of 110 μm from the retinal pigment epithelium layer to the inner-limiting membrane [36]. These steps were carried out by the Cirrus built-in software [36]. Superficial VD and vessel length density (VLD) were calculated by the built-in Cirrus 11.0 software.

SCP FI, DCP VD, VLD, and FI were analyzed in an automated fashion using *en face* angiograms in ImageJ. The ImageJ analysis started with the binarization of the images with a Li auto threshold to define the vessel signal from the background noise [37]. The auto threshold was chosen due to its repeatability, and the Li method demonstrated high concordance with previously established manual FAZ-based thresholds in our population [23,38]. VD was calculated as the percentage of the 3 × 3 mm^2^ area occupied by blood vessels. To determine VLD, the vessels were skeletonized to be 1 pixel wide using the AnalyzeSkeleton plug-in [39], and the percentage of the macular area composed of skeletonized vessels was calculated. VLD determines the total length occupied by all the blood vessels. Since all vessels are skeletonized to the same width, this parameter eliminates the disproportionate influence of larger vessels on VD. FI was calculated as the average decorrelation value of all pixels above the noise threshold, which is an indicator of blood flow velocity based on motion contrast [40].

### 2.4. Statistical Analysis

All statistical analyses were performed with R version 4 [41]. Traditional descriptive methods were used to summarize the demographic and clinical characteristics of the study participants. Multivariable linear regression models were created, regressing age against OCTA parameters while adjusting the estimates for potential confounders: race/ethnicity, sex, signal strength, presence of hypertension, and axial length for all participants, as well as duration of disease and HbA1c values in the diabetic patients. An interaction term between age and diabetes status was introduced in the analysis to model the effect of diabetes on the relationship between age and the OCTA parameters. The Benjamini–Hochberg adjustment of false discovery rate was used to adjust the *p*-values of all regressions for the presence of multiple comparisons. A value of <0.05 denoted significance for all regressions after the Benjamini–Hochberg adjustment.

## 3. Results

**Figure 1:**
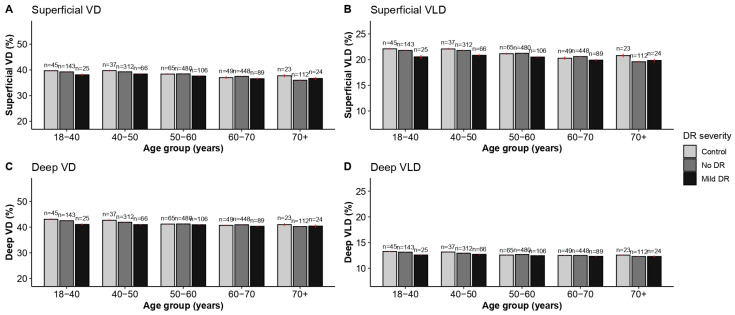
Bar plot depicting the means (±standard errors) of the superficial and deep capillary plexus vessel density and vessel length density in controls, DM no DR, and Mild DR patients as stratified by the age groups. In each of the three groups, there was a noticeable decline in vessel density metric after the fourth decade of life (*p* < 0.05 via multivariable linear regression models; see Supplemental Table S1 for the beta coefficients for age). DR = diabetic retinopathy, VD = vessel density, and VLD = vessel length density.

The patient characteristics and demographic information are shown in Table 1. This study included 217 non-diabetic, 1352 DM without DR, and 286 mild NPDR patients. The interclass correlation for grading of the FAZ parameters was >0.9 between the two graders. Figure 1 depicts that, overall, vessel density parameters decreased with increasing age, irrespective of the stage of DR. Table 2 shows the results of the multivariate regression analysis between age and the OCTA parameters in the non-diabetic and diabetic groups after adjusting for the covariates of sex, race/ethnicity, hypertension, signal strength, and axial length in all groups and, additionally, hemoglobin A1c and duration of disease in the diabetic groups. The FAZ parameters did not significantly change with increasing age in all three groups. Both superficial and deep vessel density-related parameters decreased significantly in all groups in association with increased age (Table 2).

Decreased superficial VD and VLD were associated with increased age in the non-diabetic, DM without DR, and NPDR groups (VD β *=* −0.031, −0.051, and −0.056; *p* = 0.027, <0.001, and <0.001, respectively; VLD β = −0.025, −0.036, and −0.029; *p* = < 0.01, < 0.001, and <0.001, respectively). The deep vessel density analysis in Table 2 showed that among the non-diabetics, increased age was associated with reduced DCP VD (β = −0.058, *p* < 0.001) and VLD (β = −0.20; *p* < 0.001). Among the DM no DR and the mild NPDR groups, age was also associated with reduced DCP VD (β = −0.059 and −0.41, *p* < 0.001, respectively) and VLD (β = −0.022 and −0.012, *p* < 0.001, respectively). Superficial and deep flow decreased significantly in the DM no DR (SCP FI β = −0.001, *p* < 0.01; DCP (β = <−0.001, *p* < 0.001) and mild NPDR groups with age (SCP and DCP FI β = −0.001, *p* < 0.01).

**Figure 2:**
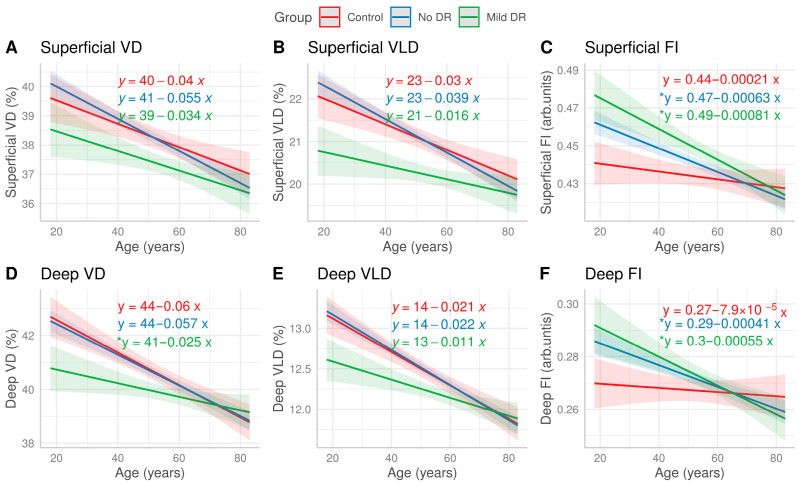
Linear regression models of the OCT angiography parameters with age, stratified by DR severity. The shaded ribbon around each regression line represents a 95% confidence interval. The equations of the regression lines are also listed for each DR severity group. All models were adjusted for the covariates of sex, hypertension, signal strength, race/ethnicity, and axial length. Overall, the Deep VD/VLD of the mild DR patients started off lower than the other two groups. However, the magnitude of associated decline with age was smaller compared to the other two groups (**D**,**E**). This trend difference was statistically significant in the Deep VD as denoted by * alongside the equation and borderline significant in the Deep VLD (*p* = 0.06). In contrast, the Superficial and Deep FI of the DM no DR and mild DR groups started off higher, but their magnitude of associated decline with age was greater compared to that of the controls (**C**,**F**). No significant differences were found in the superficial macular vasculature between the three groups (**A**,**B**).

To explore whether the association of age with OCTA parameters differed among the diabetics and non-diabetics, we then combined the diabetic and non-diabetic populations and performed a multivariable analysis that included an age by diabetes status interaction term in addition to adjusting for sex, race/ethnicity, hypertensive status, SSI, and axial length (Table 3). The interaction was found to be statistically significant in the DCP vessel density in the mild NPDR vs. control group, demonstrating that the DCP VD in the NPDR patients was not explained solely by the DR severity or by the age variable alone. The interaction term was also statistically significant for superficial and deep FI in both the DM no DR and mild NPDR cohorts, as summarized in Table 3. Table 3’s results are further visualized in Figure 2, which shows that the slope of the association between age SCP and DCP FI was statistically significantly different between the diabetic and non-diabetic patients, even though FI started higher in the younger diabetic patients (Figure 2C,F).

## 4. Discussion

In this study, we investigated the association between aging and various OCTA parameters previously known to correlate with VA [42,43] or the severity of DR [20,23,44,45]. Overall, our results show that increasing age is associated with a decrease in VD and FI among patients with no DR or mild NPDR. We also found that the association between aging and FI or DCP VD may have been modified by the presence of diabetes, such that increasing age was associated with a faster decline in FI or a slower decline in vessel density parameters in diabetics when compared to the trends in the non-diabetic patients.

Vessel density and blood flow are interrelated metrics that assess the perfusion of the retina, which is critical for its high metabolic needs [11]. VD measures the macular area covered by the vessels, thereby giving researchers a proxy value of retinal perfusion. Several studies have found VD to decrease with increasing age among healthy participants [27,28,30,46,47,48], consistent with our findings of decreased SCP and DCP VD and VLD among the controls, DM, no DR, and mild NPDR groups. As shown in Figure 1, the drop in SCP and DCP VD/VLD was most noticeable after the fourth decade of life in all three groups, a trend reported in other healthy cohorts as well [28,48,49,50]. The exact reason for this phenomenon is unknown but may be related to an accumulation of oxidative and shear stresses with age, which is associated with endothelial cellular senescence [51,52,53,54], potentially leading to vascular dysfunction.

Alterations in SCP and DCP blood flow speed have previously been reported during the early [20,22,44,55] and late [20,56] stages of DR; however, the impact of aging on blood flow in healthy [46,57,58] and diabetic people [55,59,60] is sparsely investigated. FI is a metric that allows us to estimate relative retinal perfusion by measuring the brightness of the pixel within the vessels themselves, which stands as a surrogate for blood flow velocity [40]. Interestingly, as Table 2 and Figure 2 show, SCP and DCP FI did not change significantly with increasing age among the control participants despite a concomitant reduction in SCP and DCP VD/VLD, whereas in the DM no DR and mild NPDR groups, SCP and DCP FI decreased significantly with increasing age. These disparate findings may have resulted from dysfunction of the retinal neurovascular unit (NVU)—an intricately coordinated web of interactions between the neurosensory retina, microglia, and vasculature required to maintain retinal homeostasis [61,62,63]. Damage to the NVU has recently been thought to precede vasculopathy in DR [64]. Clinical [6,59,60] and experimental studies [65,66,67] have shown that blood flow alterations may occur in the subclinical stages of DR, with mixed results in determining the direction of the altered blood flow [64]. Changes in the NVU include detachments between pericytes and basement membrane and retraction of microglial cells from the outer vasculature, disrupting the functionality of the NVU [68]. Figure 2 shows that although FI started higher in the younger patients with DM, the negative slope of the association was greater across increasing age than in the controls. Previously, studies have indicated that increased blood flow in subclinical DR compared to controls is likely a compensatory mechanism to maintain retinal perfusion due to capillary dropout [55,69]. Yet, in our study, there was no significant difference in VD/VLD reduction between the controls and the DM no DR group. Consistent with the NVU pathology paradigm, subclinical DR has been associated with microscopic changes in the vascular smooth muscle cells, pericytes, and endothelial cells, such as redistribution and changes of expression in contractile proteins in the aforementioned cell types [64]. Therefore, in such context, our results suggest that insults to the NVU may potentially lead to an altered hemodynamic autoregulatory response [61,70], which was initially reflected as an increase in FI amongst the diabetics compared to the controls. It has been hypothesized that with increasing age, this compensatory mechanism breaks down with the accumulation of neurovascular insults from diabetes [52]. This may also explain the seemingly contradictory findings of blood flow changes in other clinical studies in diabetics, which did not account for age [55,69,71,72,73]. The pathological underpinnings of the interaction between age and NVU pathology are unknown, as the concept of NVU dysfunction in subclinical DR is still nascent and is the subject of recent investigations [52,63,64,74]. Our study adds to a growing consensus that retinal blood flow may be the most sensitive OCTA metric to distinguish between the early stages of DR and needs to be investigated further as a biomarker to guide individual-specific composite risk of progressive DR [69,75,76]. As artificial intelligence and deep learning seek to become integrated in clinical workflows to screen and monitor retinal vasculature health using OCTA [77,78], our study highlights the importance of individualized screening protocols, which may utilize demographic factors and the interaction in-between to risk stratify individual patients for appropriate screening intervals and potentially early interventions. Moreover, our study points towards the possibility of using patients’ own baseline data and mapping out the trend of various parameters such as FI and VD to further offer personalized anticipatory guidance regarding the direction in which their disease pathology may be headed. This data-informed approach to offer personalized management strategies at subclinical DR may dictate patient’s willingness to participate in early interventions and risk-modifying behavior, which could potentially be vision-saving.

In addition to the interaction between FI and age, a surprising result was the smaller magnitude of decline in DCP VD with increasing age in the mild NPDR group compared to the controls (Table 3). This paradoxical result may have been because of morphological changes in the vasculature of the NPDR patients such as vascular dilation and microaneurysms, a phenomenon previously described in the literature in mild NPDR cohorts [69,79]. It is possible that in mild NPDR, a greater degree of the impairment of autoregulation with increasing age may be due to a loss of the associated compensatory mechanisms [52,54], which manifests as an apparent slower decline in vessel density despite the ongoing capillary dropout, whereas the controls and DM no DR patients did not possess this feature of pathologic vascular dilation. Interestingly, no significant change was found in the FAZ area and perimeter with increasing age. This may have been due to a high degree of interindividual variation in the FAZ parameters [11]. Results evaluating the change in FAZ area and perimeter in healthy subjects with increasing age have been mixed [6,7,29,48,80]. Some studies found a significant increase in FAZ area with age [7,48,80], whereas others have found none [6,29]. It is likely that the differences in methodologies, for example, using an SCP *en face* image instead of full thickness image, and differences in automatic versus manual grading, overshadow any real change that may occur in these parameters with aging.

The strengths of our study include a robust sample size from an urban, diverse population, stringent image quality control with manual image review, and an SSI criterion of ≥8. Our models adjusted for the confounding variables of sex, race/ethnicity, hypertension status, and axial length. The limitations of our study include the acknowledgment of the serial, cross-sectional nature of the study. A more robust design would evaluate age-associated changes in OCTA parameters within participants longitudinally—which is an ongoing investigation by our group. The recruitment of diabetic and non-diabetic controls from two different pools, i.e., a tele-medicine retinal screening program and an optometry clinic, respectively, may introduce selection bias. However, both pools are situated within the same hospital and derive from the same local patient population. Additionally, we recognize that adding HbA1c as a confounder would constitute a more inclusive model for the control participants in order to completely rule out their pre-diabetic status. However, this was not feasible in our study since a majority of the control participants did not have HbA1c documented in their medical records. Manual chart reviews were thoroughly conducted, and any borderline diabetic patients were excluded. We also note that the decorrelation values used to calculate FI were collinear with the blood flow speed within a certain range, after which blood flow speed saturated and may have depended on other factors such as vessel size. This may have limited our estimates of blood velocity in relative rather than absolute terms. Additionally, the OCTA instrument and the choice of thresholding algorithm to distinguish vessels from background noise have the potential to alter results, which is an important factor to consider for the reproducibility of studies [81,82]. The use of the Li auto thresholding algorithm means there may have been variability in translating the findings in a one-to-one fashion to other studies that use a different, well-established algorithm.

Overall, our investigation reinforced the conclusions reached in previous healthy cohorts that the OCTA parameters of VD/VLD decreased with age in controls and diabetic patients. Additionally, our study highlighted an important interaction between age and diabetes status, which impacts retinal flow index and vessel density. This finding supports the emerging idea that in subclinical retinopathy, changes in blood flow may be the earliest indicators of neurovascular damage [52,69,83]. Our study indicates that age may be a crucial factor to account for when elucidating the connection between neurovascular damage and autoregulation, especially during the search for appropriate OCTA markers of early pathology.

## Figures and Tables

**Table 1:** Demographic and image characteristics of the study participants.

Diabetic Retinopathy Severity				
Characteristic	Control, *n* = 217	No DR, *n* = 1352	Mild DR, *n* = 286	Total, *n* = 1855
Age (years), mean (SD)	53 (14)	55 (11)	56 (11)	55 (11)
Age group (years), *n* (%)				
18–40	45 (21)	130 (9.6)	22 (7.7)	197 (11)
40–50	37 (17)	271 (20)	61 (21)	369 (20)
50–60	64 (29)	442 (33)	98 (34)	604 (33)
60–70	49 (23)	409 (30)	83 (29)	541 (29)
70+	22 (10)	100 (7.4)	22 (7.7)	144 (7.8)
Sex, *n* (%)				
Male	101 (47)	660 (49)	148 (52)	909 (49)
Female	116 (53)	692 (51)	138 (48)	946 (51)
Race/ethnicity, *n* (%)				
Hispanic	79 (36)	555 (41)	100 (35)	734 (40)
NH Asian	51 (24)	352 (26)	82 (29)	485 (26)
NH Black	14 (6.5)	88 (6.5)	25 (8.7)	127 (6.8)
NH White	48 (22)	117 (8.7)	24 (8.4)	189 (10)
Other	25 (12)	240 (18)	55 (19)	320 (17)
Presence of hypertension, *n* (%)	76 (35)	800 (59)	179 (63)	1055 (57)
Duration of diabetes (years), mean (SD)	-	6 (6)	10 (7)	7 (6)
Hemoglobin A1c (%), mean (SD)	-	7.87 (3.45)	8.61 (1.99)	8.00 (3.25)
Axial length (mm), mean (SD)	23.60 (1.11)	23.49 (0.98)	23.46 (1.00)	23.50 (1.00)
LogMAR, mean (SD)	0.04 (0.07)	0.09 (0.12)	0.09 (0.10)	0.08 (0.11)
Signal strength, mean (SD)	9.76 (0.56)	9.76 (0.54)	9.76 (0.51)	9.76 (0.53)

DR = diabetic retinopathy, NH = non-Hispanic, SD = standard deviation, mm = millimeter.

**Table 2:** Multivariate regression analysis of the association between age and the optical coherence tomography angiography (OCTA) parameters.

	Control (*n* = 217)		DM No DR (*n* = 1352)		Mild NPDR (*n* = 286)	
Parameter	Estimate (95% CI)	*p*-Value	Estimate (95% CI)	*p*-Value	Estimate (95% CI)	*p*-Value
FAZ area (mm^2^)	<0.001 (−0.001, 0.001)	0.777	<0.001 (−0.000, 0.001)	0.171	<0.001 (−0.001, 0.001)	0.898
FAZ perimeter (mm)	0.002 (−0.003, 0.007)	0.567	0.002 (−0.000, 0.004)	0.121	−0.002 (−0.008, 0.004)	0.627
Superficial VD (%)	−0.031 (−0.056, −0.007)	0.026 *	−0.050 (−0.062, −0.038)	<0.001 ***	−0.056 (−0.084, −0.028)	<0.001 ***
Superficial VLD (%)	−0.025 (−0.041, −0.010)	0.003 **	−0.036 (−0.043, −0.028)	<0.001 ***	−0.029 (−0.047, −0.011)	0.002 **
Superficial FI	>−0.001 (−0.001, 0.000)	0.343	−0.001 (−0.001, −0.001)	<0.001 ***	−0.001 (−0.001, >−0.001)	<0.001 ***
Deep FI	>−0.001 (−0.000, 0.000)	0.774	>−0.001 (−0.001, −0.000)	<0.001 ***	−0.001 (−0.001, >−0.001)	<0.001 ***
Deep VD (%)	−0.051 (−0.070, −0.033)	<0.001 ***	−0.055 (−0.064, −0.045)	<0.001 ***	−0.052 (−0.072, −0.032)	<0.001 ***
Deep VLD (%)	−0.018 (−0.024, −0.013)	<0.001 ***	−0.022 (−0.025, −0.019)	<0.001 ***	−0.017 (−0.025, −0.009)	<0.001 ***

* *p* < 0.05, ** *p* < 0.01, *** *p* < 0.001 (Benjamini–Hochberg adjusted). The values very close to zero are reported as ‘<0.001’ for positive values or ‘>−0.001’ for negative values. Each estimate denotes the change in parameter value per year of increase in age. A visual representation of these estimates can be observed in Supplemental Figure S1. All analysis adjusted for the covariates of sex, race/ethnicity, hypertension, signal strength, and axial length. Analysis in the DM no DR and mild NPDR groups additionally adjusted for hemoglobin A1c and duration of disease. Abbreviations: CI—confidence interval, mm –millimeter, μm—micrometer, SCP—superficial capillary plexus, DCP—deep capillary plexus, FAZ—foveal avascular zone, VD—vessel density, VLD—vessel length density, FI—flow index, DR—diabetic retinopathy, and NPDR—non-proliferative diabetic retinopathy.

**Table 3:** Multivariate regression analysis for the association between age and the optical coherence tomography angiography (OCTA) parameters as modified by the presence of diabetes severity in patients with and without diabetes.

	Age × DM No DR			Age × Mild NPDR		
	Estimate	95% CI	*p*-Value	Estimate	95% CI	*p*-Value
FAZ area (mm^2^)	<0.001	(−0.001, 0.001)	0.797	−0.001	(−0.002, 0.001)	0.521
FAZ perimeter (mm)	<0.001	(−0.005, 0.005)	0.924	−0.005	(−0.011, 0.002)	0.240
SCP VD (%)	−0.015	(−0.039, 0.009)	0.301	0.006	(−0.025, 0.038)	0.793
SCP VLD (%)	−0.009	(−0.024, 0.006)	0.312	0.014	(−0.006, 0.034)	0.240
SCP FI	>−0.001	(−0.001, 0)	0.027 *	>−0.001	(−0.001, 0)	0.015 *
DCP FI	>−0.001	(−0.001, 0)	0.030 *	>−0.001	(−0.001, 0)	0.025 *
DCP VD (%)	0.003	(−0.018, 0.025)	0.797	0.035	(0.007, 0.063)	0.029 *
DCP VLD (%)	0.003	(−0.018, 0.025)	0.797	0.035	(0.007, 0.063)	0.059

* Statistical significance at *p* < 0.05 after Benjamini–Hochberg multiple comparison adjustment. The values very close to zero are reported as ‘<0.001’ for positive values or ‘>−0.001’ for negative values. All analysis adjusted for the covariates of sex, race/ethnicity, hypertension, signal strength, and axial length. Abbreviations: CI—confidence interval, mm–millimeter, μm—micrometer, SCP—superficial capillary plexus, DCP—deep capillary plexus, FAZ—foveal avascular zone, VD—vessel density, VLD—vessel length density, FI—flow index, DR—diabetic retinopathy, and NPDR—non-proliferative diabetic retinopathy.

## Data Availability

The original contributions presented in this study are included in the article and Supplementary Material. Further inquiries can be directed to the corresponding author.

## Supplementary Materials

The following supporting information can be downloaded at: https://www.mdpi.com/article/10.3390/jpm16060286/s1, Figure S1: Effect of Age on OCTA parameters; Table S1: Beta coefficients for age amongst diabetic and non-diabetic patients.
