# Supplementary material for: Diabetes May Modulate the Association Between Age and Optical Coherence Tomography Angiography Parameters: A Serial, Cross-Sectional Study
Source: J Pers Med. 2026 May 26;16(6):286. doi: 10.3390/jpm16060286 (PMC13302122; doi:10.3390/jpm16060286)
Supplement: Supplementary file 1 [file jpm-16-00286-s001.zip › jpm-4260249-supplementary.pdf]

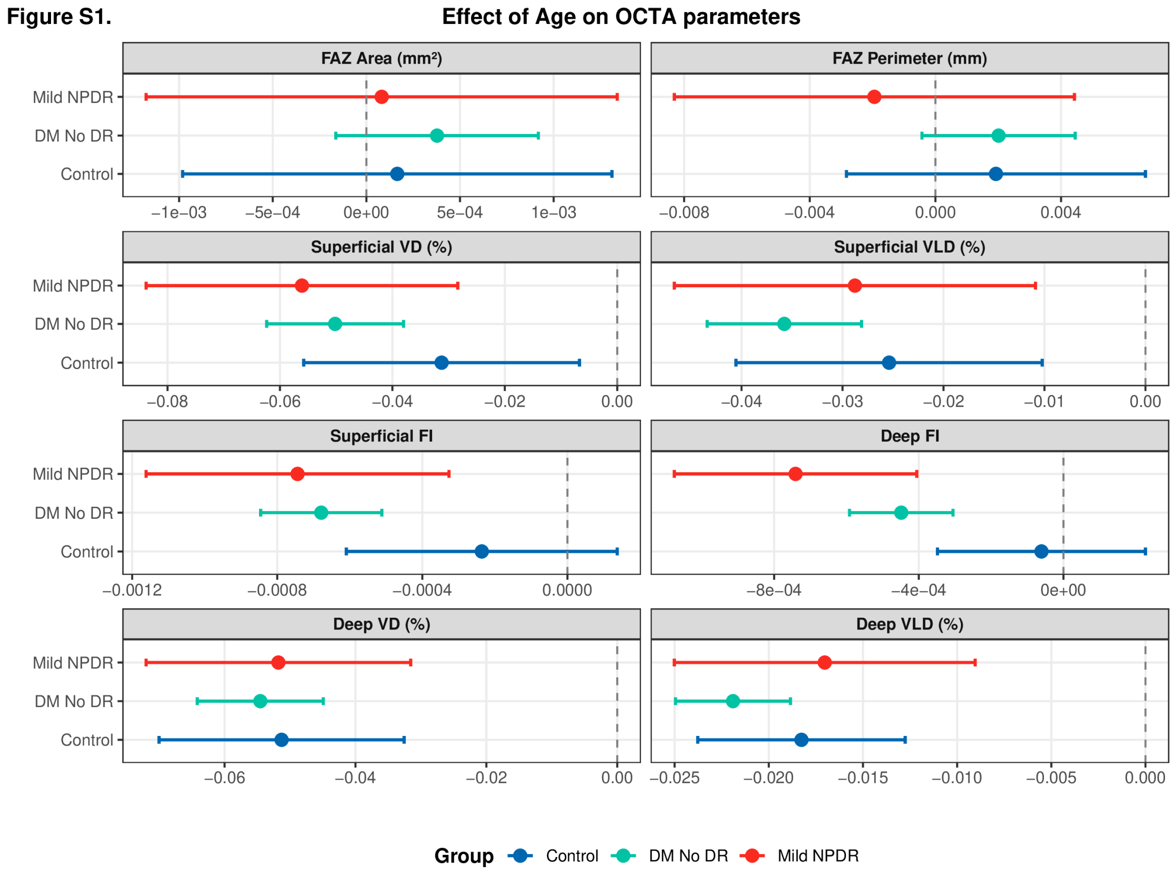

Figure S1. Effect of Age on OCTA parameters.

Table S1. Beta coefficients for age amongst diabetic and non-diabetic patients.

Diabetics			Non diabetics		
**Parameter**	**Beta coefficient for age**	**P value**	**Parameter**	**Beta coefficient for age**	**P value**
SUPERFICIAL VD (%)	-0.049269653	9.79136E-18	SUPERFICIAL VD (%)	-0.031248079	0.012794087
SUPERFICIAL VLD (%)	-0.032989532	1.36332E-19	SUPERFICIAL VLD (%)	-0.025386852	0.001135991
Deep VD (%)	-0.052649684	2.19789E-31	Deep VD (%)	-0.051284287	1.81425E-07
Deep VLD (%)	-0.020574005	1.8711E-42	Deep VLD (%)	-0.018263868	4.75106E-10

VD = vessel density, VLD = vessel length density.
